# Oxaliplatin-induced lung fibrosis

**DOI:** 10.4103/0971-5851.64259

**Published:** 2009

**Authors:** Arpan Shah, Zarir F. Udwadia, Sachin Almel

**Affiliations:** *Departments of Chest Medicine, P.D. Hinduja Hospital, Veer Savarkar Marg, Mahim (W), Mumbai, India*; 1 *Departments of Oncology, P.D. Hinduja Hospital, Veer Savarkar Marg, Mahim (W), Mumbai, India*

**Keywords:** Drug toxicity, lung fibrosis, oxaliplatin

## Abstract

Oxaliplatin has been approved for use as an adjuvant treatment in stage III colorectal carcinoma by the US-FDA. The majority of toxicity caused by this drug is manageable. However, rare, isolated cases of pulmonary fibrosis induced by this drug have been reported in literature. We report one such case of rapidly evolving pulmonary fibrosis following treatment with oxaliplatin.

## INTRODUCTION

Oxaliplatin [ELOXATIN], a third-generation platin derivative, acts via interruption of DNA synthesis.[1] Its use, along with fluoropyrimidines, has shown a survival benefit in stage III colorectal adenocarcinoma, achieving a disease-free survival rate of 78.2% at three years.[2–3] Oxaliplatin, therefore, represents the first choice adjuvant treatment in this group of patients. Pulmonary toxicity is a very rare side effect. We report a case of fatal pulmonary toxicity in a patient on oxaliplatin.

## CASE REPORT

A 76 year old gentleman presented with a mass in the right lower quadrant of his abdomen, since six months. It was associated with loss of appetite and weight loss of approximately 5–7 kilograms. There was no history of diarrhea, vomiting, cough or breathlessness. A routine ultrasound of the abdomen revealed a well defined, echogenic mass (approximately 17 cm × 5.2 cm × 8.5 cm) in the right iliac fossa. In view of this, a CT scan of the abdomen was taken, which confirmed a large multiloculated mass in the right iliac fossa involving the cecum, appendix, and terminal ileum, with multiple right renal calculi and cortical rearing with a cystic lesion in the right lobe of the liver. Colonoscopy suggested a polypoid friable growth with ulceration in the cecum. Biopsy from the cecal mass showed a well differentiated adenocarcinoma.

**Figure 1:**
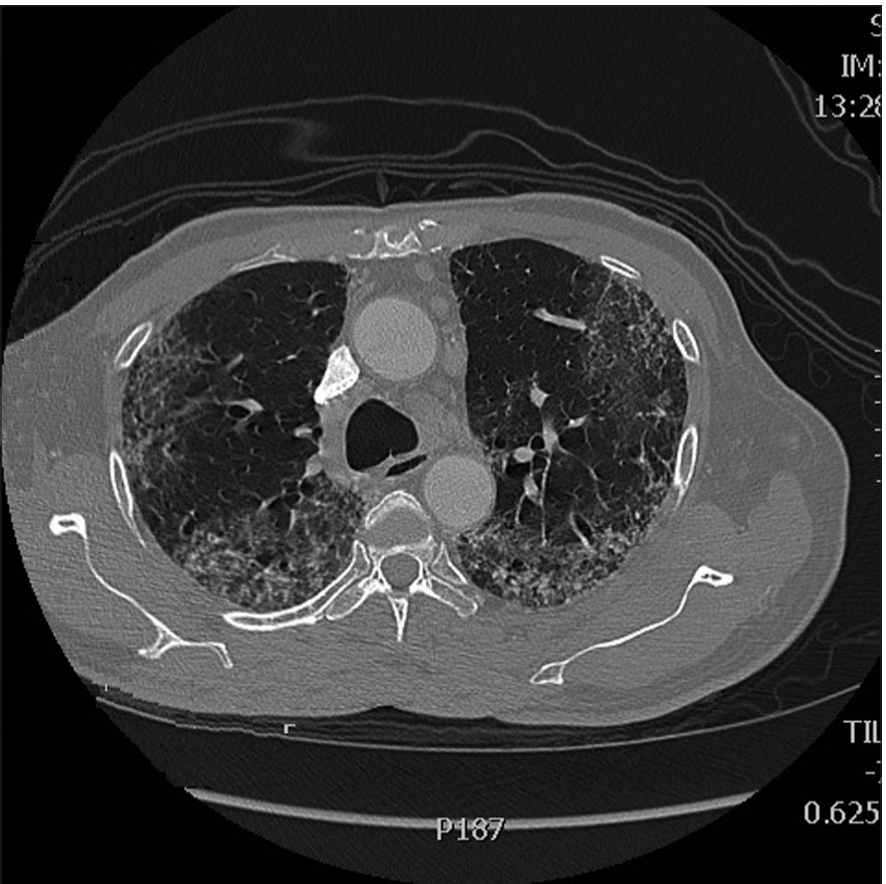
High resolution computed tomography scan 1

**Figure 2:**
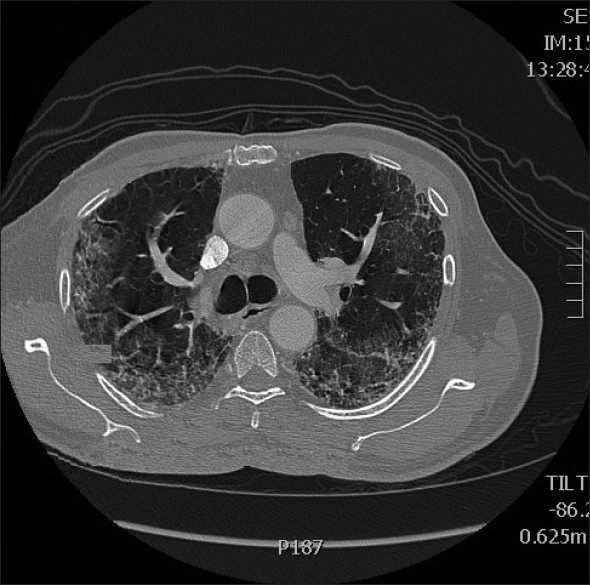
High resolution computed tomography scan 2

Adjuvant chemotherapy with oxaliplatin given at a dose of 130 mg/m^2^ intravenously on Day 1 of each three-week cycle, and capecitabine at a dose of 2000 mg/m^2^/day orally in two divided doses was given on Days 1–14 of each cycle.[4] The patient tolerated the initial cycle well; however, after the second cycle of the treatment, which was three weeks later, he developed progressive dyspnea and persistent cough despite having no history of any previous lung disease, and a pre-treatment chest X-ray was normal. A chest radiograph revealed diffuse bilateral interstitial infiltrates and an ABG performed showed significant hypoxia with PaO_2_ of 55 mmHg. A high resolution computed tomography (HRCT) scan [Figures 1 and 2] showed bilateral alveolar infiltrates predominant in the bases. A trans-bronchial lung biopsy could not be performed as he was extremely breathless and hypoxic. He was commenced on 80 mg of methylprednisolone thrice a day. However his clinical condition began to deteriorate and within a week of starting steroids he succumbed to his illness.

## DISCUSSION

Oxaliplatin has become part of the standard treatment regime in a high number of patients with colorectal cancer in both the advanced and early stages. Early safety studies have found no significant increase in pulmonary complications, except for the dyspnea that may have occurred in the setting of a hypersensitivity reaction.[5–6] Not much data is available regarding its pulmonary toxicity, although few isolated case reports of respiratory insufficiency associated with pulmonary infiltrates evolving to pulmonary fibrosis have been documented.[7–10] As no other causative factor leading to respiratory insufficiency can be found in these patients, the condition has therefore been attributed to oxaliplatin. Our patient had a rapidly progressive respiratory insufficiency that was refractory to conventional medical treatment. No infectious causes of his clinical picture could be identified.

Although there is no objective evidence correlating oxaliplatin to the direct damage of lung parenchyma, there are data suggesting that oxaliplatin may cause glutathione depletion, which may be involved in the pathogenesis of liver damage caused by the drug, which may be related to endothelial and perivascular damage and obstruction of the hepatic sinusoids.[11] Glutathione is a small antioxidant molecule. In the lung, glutathione plays a significant role as a protector against oxidative damage, and the depletion caused by oxaliplatin may be the factor triggering the pulmonary lesions, leading to interstitial pneumonitis and subsequent pulmonary fibrosis. There is no adequate data on whether treatment with antioxidant agents may be beneficial for these patients. Treatment with N-acetylcysteine, which may replenish glutathione deposits, may probably have some value.

Most cases of pulmonary fibrosis associated with oxaliplatin, reported in literature, have a rapid and fatal course[7–10] similar to our patient. Of late, all these studies have utilized oxaliplatin in combination with 5FU, Leucoverin or capecitabine either singly or in combination.[7–10] Fibrosis is known to occur in some patients using oxaliplatin with 5FU and Leucoverin as a regimen and none reported so far in regimens using capecitabine.[12] However, it is not known whether prior interstitial lung damage or impairment in respiratory function tests may make development of oxaliplatin-induced interstitial pneumonitis more likely,[13] although a pre-treatment routine pulmonary function test along with diffusing capacity may guide us.

Therapy with corticosteroids led to a short improvement; however, the patient died one week after the initiation of corticosteroid treatment, due to respiratory insufficiency. The clinical and radiological findings as well as the lack of an infectious cause indicate that pulmonary fibrosis was induced by oxaliplatin.

## CONCLUSION

Our case demonstrates that although pulmonary fibrosis is rare as compared to other infectious pulmonary complications in patients treated with oxaliplatin, a high index of suspicion has to be maintained in the clinical course of an otherwise unexplained pulmonary infiltrate, in order to prevent significant morbidity and mortality.
